# 2900. Clinical Utility of Repeat Magnetic Resonance Imaging Studies Among Children with Acute Hematogenous Osteomyelitis

**DOI:** 10.1093/ofid/ofad500.171

**Published:** 2023-11-27

**Authors:** Abby Thorne, Angela Moss, Julia S Sanders, Jill Stein, Justin B Searns

**Affiliations:** University of Colorado School of Medicine, Denver, CO; University of Colorado School of Medicine,Adult and Child Center for Health Outcomes Research and Delivery Science, Aurora, Colorado; University of Colorado, Department of Orthopedic Surgery, Aurora, Colorado; University of Colorado, Department of Radiology, Aurora, Colorado; Children’s Hospital Colorado, University of Colorado, Aurora, Colorado

## Abstract

**Background:**

Magnetic resonance imaging (MRI) is the diagnostic study of choice for children with acute hematogenous osteomyelitis (AHO). However, there is limited guidance for whether repeat MRIs are clinically impactful for children with AHO who fail to improve after initial treatment. This study aimed to determine whether repeat MRIs changed clinical management among children with AHO.

**Methods:**

Children admitted to a quaternary care pediatric hospital with a discharge diagnosis of osteomyelitis were identified during a 9-year period. Patients with chronic symptoms, non-hematogenous infections, or significant contributing comorbidities were excluded. Medical records were retrospectively reviewed for all MRIs performed 3 weeks prior to admission to 24 months after discharge. An MRI was considered clinically impactful if it identified a new infectious process (e.g., abscess not seen on initial MRI) or if it resulted in surgical intervention within 24 hours. Categorical variables were compared using Fisher’s Exact or chi-square tests and continuous with Wilcoxon Rank-Sum tests.

**Results:**

Among 239 included patients, 41 (17%) had more than 1 MRI performed during clinical care for their AHO. Twenty-nine repeat MRIs occurred during initial hospitalization and 27 during outpatient follow up. Children who underwent repeat inpatient MRIs had longer hospitalizations (9 vs 5 days, P< 0.01) and higher C-reactive protein levels (20 vs 7 mg/dL, P< 0.01), and were more likely to be critically ill (20% vs 6%, P=0.02) or to experience a therapeutic failure (20% vs 3%, P< 0.01). Among repeat inpatient MRIs, 19 (66%) were clinically impactful; 9 (31%) led to repeat surgical procedures and 13 (45%) identified new diagnoses. Repeat outpatient MRIs were more likely to be performed in children with vertebral AHO or multifocal infections (P=0.02). Among repeat outpatient MRIs, 7 (25%) were clinically impactful. Six (22%) led to repeat operative procedures and 3 (11%) identified new diagnoses.

**Figure:**
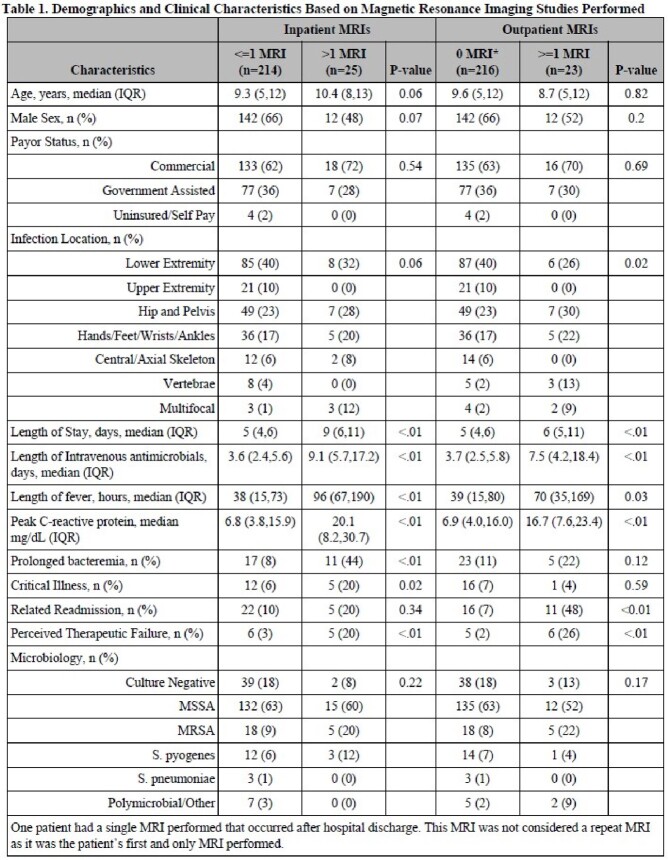

**Conclusion:**

When used judiciously among ill children with complicated AHO, repeat MRI can be clinically impactful in both the inpatient and outpatient setting. Prospective studies are needed to better define which children with AHO benefit from repeat MRI.

**Disclosures:**

**Julia S. Sanders, MD**, OrthoPediatrics Corp: Advisor/Consultant

